# Hypoxia Promotes Epithelial - Mesenchymal Transition of Hepatocellular Carcinoma Cells via Inducing GLIPR-2 Expression

**DOI:** 10.1371/journal.pone.0077497

**Published:** 2013-10-29

**Authors:** Shao-guang Huang, Le-le Zhang, Qin Niu, Gui-ming Xiang, Lin-lin Liu, Dong-neng Jiang, Fei Liu, Yi Li, Xiaoyun Pu

**Affiliations:** 1 Department of Clinical Laboratory, Xin Qiao Hospital, Third Military Medical University, Chong Qing, People's Republic of China; 2 Institute of Pathology, Southwest Hospital, Third Military Medical University, Chong Qing, People's Republic of China; Vanderbilt University Medical Center, United States of America

## Abstract

Glioma pathogenesis related-2 (GLIPR-2) belongs to pathogenesis related-1 (PR-1) family whose function remains unknown. In our previous studies, GLIPR-2 was found to be a novel potent stimulator of epithelial-to-mesenchymal transition (EMT) in renal fibrosis which has been classified as type 2 EMT. However, whether GLIPR-2 could induce type 3 EMT in carcinogenesis needs further investigation. In this study, we showed that GLIPR-2 was expressed in hepatocellular carcinoma (HCC) tissues, hypoxia could upregulate the expression of GLIPR-2 in HepG2 and *PLC*/PRF/5 cells in vitro, overexpression of this protein promoted migration and invasion via EMT, knockdown of GLIPR-2 attenuated migration and invasion of HepG2 and *PLC*/PRF/5 cells in hypoxia. Moreover, extracellular signal-regulated kinases 1 and 2 (ERK1/2) are positively regulated by GLIPR-2. Taken together, we provide evidence for a hypoxia/GLIPR-2/EMT/migration and invasion axis in HCC cells and it provides novel insights into the mechanism of migration and invasion of hepatocellular carcinoma cells in hypoxia condition.

## Introduction

Primary hepatocellular carcinoma (HCC) is one of the most common and serious malignancies worldwide with poor prognosis because of invasion and metastasis in the early stage [1], [2]. Epithelial-to-mesenchymal transition (EMT) of HCC cells is a key event in cancer metastasis and lead to poor patient outcome [3], [4]. EMT is defined as a cellular process by which differentiated epithelial cells lose their epithelial phenotype and acquire characteristic features of mesenchymal cells. It was showed by in vitro assay that, during EMT, the differentiated epithelial cells lose their apical-basal polarity and epithelial adhesion (E-cadherin positive), and acquire a myofibroblast phenotype (α-smooth muscle actin and vimentin positive) accompanied by enhanced cell migration and invasion [5], [6]. In recent reviews, EMT has been classified as three different subtypes based on different biological settings which cause various functional consequences. Type 1 EMT was considered to be associated with implantation, embryogenesis, and organ development. EMT occurred in organ fibrosis was classified as type2 and EMT in carcinogenesis was classified as type 3 [7].

Hypoxia has been considered as an inducer of EMT-like process in diverse human solid malignancies such as liver cancer, breast cancer, and prostate cancer [8], [9]. In hypoxic microenvironment, cancer cell signaling pathways including PI3K/Akt signaling, Notch signaling and MAPK signaling pathway may be activated to regulate EMT that facilitate cancer invasion and metastasis [10], [11].

Glioma pathogenesis related-2 (GLIPR-2), also called GAPR-1 and C9orf19, is a conserved mammalian protein belonging to pathogenesis related-1 (PR-1) family. In humans, it is mainly expressed in peripheral leukocytes and lungs in human [12], [13]. However, the function of this gene remains to be clarified. Previous studies have shown that GLIPR-2 is abundantly increased in PTCs during kidney fibrogenesis [14]. Further more, our previous results demonstrated that GLIPR-2 contributes significantly to EMT in HK-2 cells which has been classified as type 2 EMT [15]. However, whether GLIPR-2 could induce type 3 EMT occured in carcinogenesis needs further investigation. Studies on GLIPR-2 gene regulating EMT in carcinogenesis may therefore yield important insights into the mechanisms of cancer metastasis as well as therapeutic treatment.

In this study, we provide evidence for a hypoxia/GLIPR-2/EMT/migration and invasion axis in HepG2 and *PLC*/PRF/5 cells. Hypoxia upregulated the expression of GLIPR-2 and GLIPR-2 overexpression promoted migration and invasion via EMT in vitro. Knockdown of GLIPR-2 attenuated migration and invasion of HepG2 and *PLC*/PRF/5 cells in hypoxia. Moreover, extracellular signal-regulated kinases 1 and 2 (ERK1/2) are positively regulated by GLIPR-2. Thus, we demonstrate that GLIPR-2 is induced by hypoxia and may play a central role in EMT by targeting ERK1/2 signaling pathway in HCC cells.

## Materials and Methods

### Ethics Statement

All the liver cancer pathological sections were received permission of patients. The study was carried out according to the Helsinki Declaration and the samples were processed under approval of the written consent statement by Ethical Committee of Xinqiao Hospital of Third Military Medical University, Chongqing, China.

### Cell Culture

The human HCC cell lines HepG2 and *PLC*/PRF/5 were purchased from China Center for Type Culture Collection (CCTCC). The cells of 10∼20 passages were grown in Dulbecco’s modified Eagle’s medium (DMEM/Low Glucose, Sigma-Aldrich, St. Louis, MO, USA), supplemented with 10% fetal bovine serum (FBS, GIBCO, Invitrogen, USA), 100 U/ml penicillin G, and 100 µg/ml streptomycin. Cells were grown in a humidified incubator at 37°C in an atmosphere of 5% CO2 and 95% air. For hypoxic culture, cells were placed in a hypoxic (1% O2, 5% CO2, 37°C) incubator (PrecisionScientific, Winchester, VA, UK) for 0, 24, 48, 72 h. PD98059, specific inhibitors of MEK, was purchased from Invitrogen and revolved with DMSO until the concentration was 5 mg/ml. DMSO was applied as the control.

### Plasmid Construction and Transfection

The plasmid pcDNA3.0- GLIPR-2 was constructed and preserved in our laboratory. Briefly, the full length GLIPR-2 was constructed with eukaryotic expression vector pcDNA3.0-GLIPR-2 using the two linkers: the forward primer, 5′-CGCGGTACCATGGGCAAGTCAGCATCCAAACAGT-3′ (*KpnI* site underlined); the reverse primer, 5′-GGCGAATTCTTACTTCTTCGGCGGCAGGACGTT-3′ (*EcoRI* site underlined). The results were identified by sequencing. 1×10^5^ cells were plated per well in a 6-well plate with 2 ml DMEM/Low Glucose containing 20%FBS and incubated until they reached 30–40% confluence. Then cells were transiently transfected with the plasmid pcDNA3.0- GLIPR-2 according to the protocol of Lipofectamine™ 2000 Lipofectamine transfection reagent (Invitrogen, Camarillo, CA, USA). Seventy-two hours later, the cells were performed for the following experiments. Plasmid pcDNA3.0 at identical operation was used as the control in parallel experiments.

### Stable shRNA-Expressing Cell Lines and Flow Cytometry Assay

HepG2 and *PLC*/PRF/5 cell lines were infected cells with pMagic 7.1-based lentiviral particles containing shRNAs targeted to GLIPR-2 to generate stable knockdown clones. The lentiviral GLIPR-2-shRNA constructs were designed, synthesized, and supplied by Shanghai SunBio Medical Biotechnology Co., Led. China. Two of the three effective targets were selected and sequences are CcggGATGGTACAGTGAAATCAATTCAAGAGATTGATTTCACTGTACCATCTTTTTTg and CcggGCCATGGTATGGAAGAACATTCAAGAGATGTTCTTCCATACCATGGCTTTTTTg. 24 hours post-infection, cells were propagated and preserved. After three washes, cultured cells infected with lentiviral particles were isolated and re-suspended in PBS. Subsequently, cells were analyzed using a FACScan (Becton Dickinson, Franklin Lakes, NJ,USA) and the EGFP-positive population was selected by flow cytometry. The cells infected with scrambled sequences were used as negative controls.

### RT-qPCR

1 µg total RNA was reverse-transcribed into cDNA with M-MLV reverse transcriptase (Promega, Madison, WI, USA) as the manufacturer’s directions. The following forward and reverse primers were used: GLIPR-2, 5′-ATCAAGAACTATAACTTCCAGCAGC-3′ (forward), 5′-AGCCCTCATTGACAACATTCC-3′ (reverse); β-actin, 5′-TGACGTGGACATCCGCAAAG-3′ (forward), 5′-CTGGAAGGTGGACAGCGAGG-3′ (reverse). Reaction conditions were as follows: 95°C for 30 s, 95°C for 5 s, 62°C for 10 s, and 72°C for 45 s for 40 cycles. Nuclease-free water (TIANGEN, Beijing, China) was substituted for the cDNA as a negative control in each PCR reaction. The relative gene expression was determined using the 2−ΔΔCt method according to the manufacturer’s recommended protocol.

### Western Blotting

Harvested cells were homogenized with lysis buffer and cell lysates were centrifuged at 4° for 15 minutes at 10,000 rpm. Then the supernatant was placed in fresh tubes and quantified using the Bradford protein assay. 50 µg proteins underwent electrophoresis on a 12% SDS-polyacrylamide gel were transferred to a PVDF membrane (Bio-RAD, Hercules, CA, USA) by electroblotting. After blocking with 5% fat-free milk in PBS for 2 h at room temperature, the membranes were incubated with a primary antibody overnight at 4°C. After repeated washing, the membranes were incubated with a horseradish peroxidase–conjugated secondary antibody (ZSGB Biotechnology, Beijing, China) (1∶2000) and enhanced elechemiluminescence (ECL) detecting reagent (Princeton, NJ, USA). Mouse monoclonal antibodies against E-cadherin, vimentin, α-SMA and GLIPR-2 were obtained from Santa Cruz (1∶500), and anti p44/42 or phospho-p44/42 MAPK (Erk1/2) (1∶2000) monoclonal antibodies from Cell Signaling Technology.

### Immunohistochemistry

Paraffin-embedded tissue sections were fixed in xylene for 10 min and then hydration by 100%, 95%, 90%, 85%, 80%, 75% ethanol. After blocking endogenous peroxides and proteins, slides were incubated with anti-GLIPR-2 monoclonal antibody (1∶200) diluted with PBS for 1 hours at room temperature and then incubated with horseradish peroxidase–conjugated goat-anti-rat secondary antibody for 10 min at room temperature, incubated with diaminobenzidine solution for 10 min and counterstained with hematoxylin. After dehydration by 75%, 80%, 85%, 90%, 95%, 100%, slides mounting were performed by resina.

### Migration and Invasion Assays

1×10^5^ transfected cells in serum-free DMEM were added to the top well of each migration chamber with an 8-µm pore size membrane (Millpore, Volketswil,Switzerland). 600 µl DMEM with 10% FBS was added in the bottom chambers and analyzed after 24 hours. For the invasion assays, the upper chambers were coated with ECM gel (Sigma, St. Louis, MO, USA). FBS was added to the lower chamber as described for the cell migration experiments.

### Statistical Analysis

All experiments were repeated in triplicate. Statistical analysis was performed using a one-way analysis of variance (ANOVA) and all values were expressed as means ± SD. A statistical package SPSS11.0 (SPSS Inc., Chicago, USA) was used for all analysis. *P*<0.01 was considered statistically significant.

## Results

### Hypoxia Induces GLIPR-2 Expression in Human HCC Cell Lines

**Figure 1:**
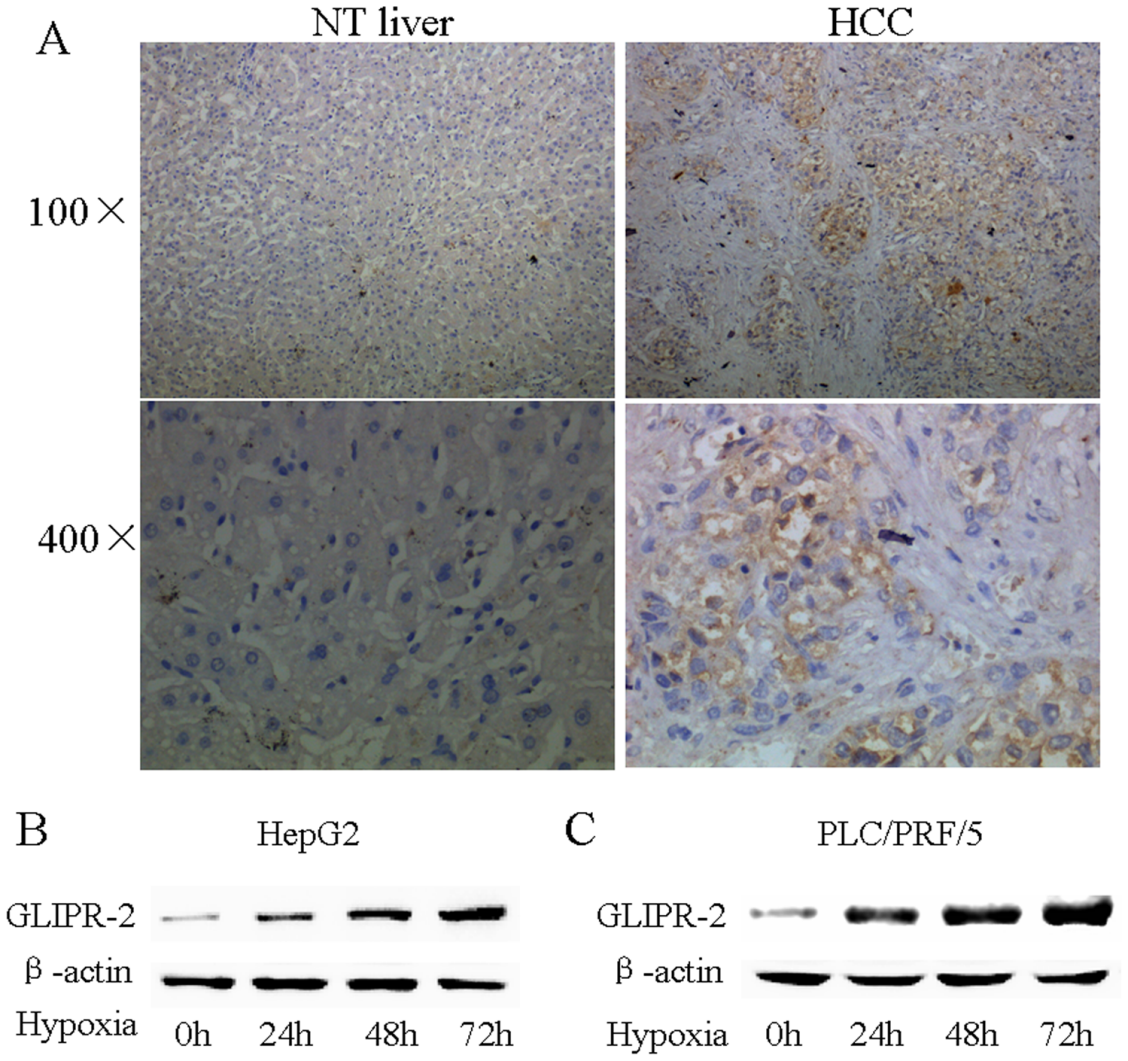
GLIPR-2 overexpressed in human HCC and cell lines in hypoxia condition. (A) Normal liver tissues had very low expression of GLIPR-2, whereas GLIPR-2 expression was increased in the cancer tissue in 2 cases. (B) GLIPR-2 expression (18 kDa) in HepG2 cells in hypoxia or normoxia condition. (C) GLIPR-2 expression (18 kDa) in *PLC*/PRF/5 cells in hypoxia or normoxia condition. The results suggest that hypoxia induces GLIPR-2 expression in HepG2 and *PLC*/PRF/5 cells in a time-dependent manner.

To determine whether GLIPR-2 is expressed in human HCC tissues, we detected 5 paired liver cancer paraffin-embedded tissue sections by immunohistochemistry against GLIPR-2. As shown in Figure 1 A, normal liver tissues had very low expression of GLIPR-2, whereas GLIPR-2 expression increased in the HCC tissues partially which may hint some kind of factor promote GLIPR-2 expression. Hypoxia is considered as a microenviroment in solid tumor result from HCC cells growing abundantly and fleetly which may responsible for GLIPR-2 expression. To confirm this hypothesis, we incubated HepG2 and *PLC*/PRF/5 cells in normoxia (21% O_2_) or hypoxic conditions (1% O_2_). As shown in Figure 1 B and C, GLIPR-2 expression was upregulated after 24 h, 48 h or 72 h of hypoxia. B, western blotting analysis of GLIPR-2 expression (18 kDa) in hypoxia condition in HepG2 cells. C, western blotting analysis of GLIPR-2 expression (18 kDa) in hypoxia condition in *PLC*/PRF/5 cells. The results suggest that hypoxia induces GLIPR-2 expression in HepG2 and *PLC*/PRF/5 cells in a time-dependent manner. Together, these results suggest that GLIPR-2 expresses in HCC tissues and can be induced by hypoxia in a time-dependent manner in HepG2 and *PLC*/PRF/5 cells in vitro.

### GLIPR-2 Overexpression Promotes Migration and Invasion via EMT through ERK1/2 Activation

**Figure 2:**
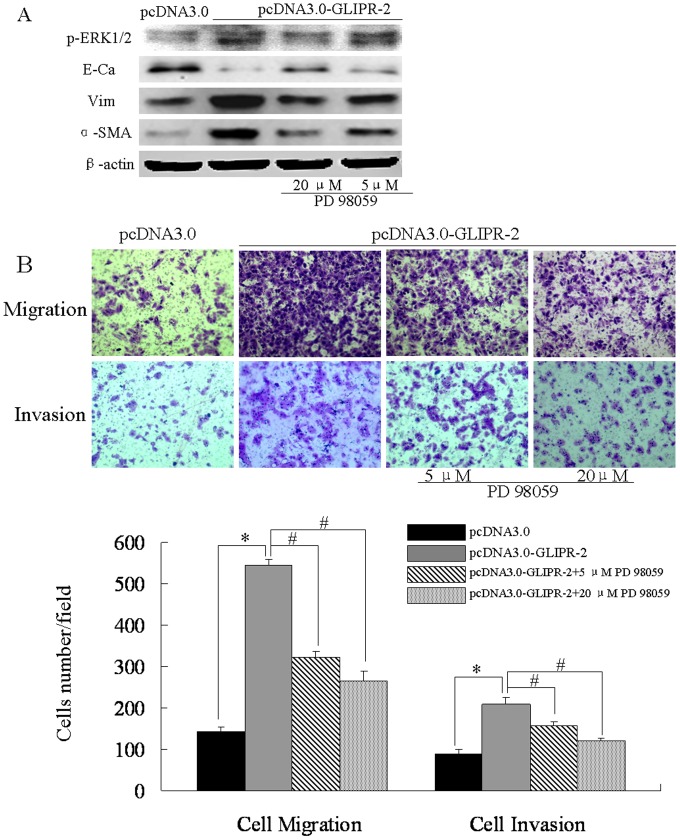
GLIPR-2 induces the EMT-like phenotype following enhanced migration and invasion of HepG2 cells via ERK1/2 activation. (A) GLIPR-2 overexpession induced the EMT-like phenotype via ERK1/2 pathway. P-ERK1/2 was elevated in pCDNA3.0- GLIPR-2 transfected HepG2 cells but decreased gradually in dose-dependently manner of PD98059. E-cadherin decreased in pCDNA3.0- GLIPR-2 transfected HepG2 cells but increased gradually in dose-dependently manner of PD98059. Vimentin and α-smooth muscle actin increased in pCDNA3.0- GLIPR-2 transfected HepG2 cells but decreased gradually in dose-dependently manner of PD98059. (B) GLIPR-2 promotes cell migration and invasion via ERK1/2 activation. Data are presented as mean ± SD. *P<0.01 compared with the pcDNA3.0 group (black bar), ANOVA. ^#^ *P*<0.01 compared with the GLIPR-2 transfection group (grey bar), ANOVA.

**Figure 3:**
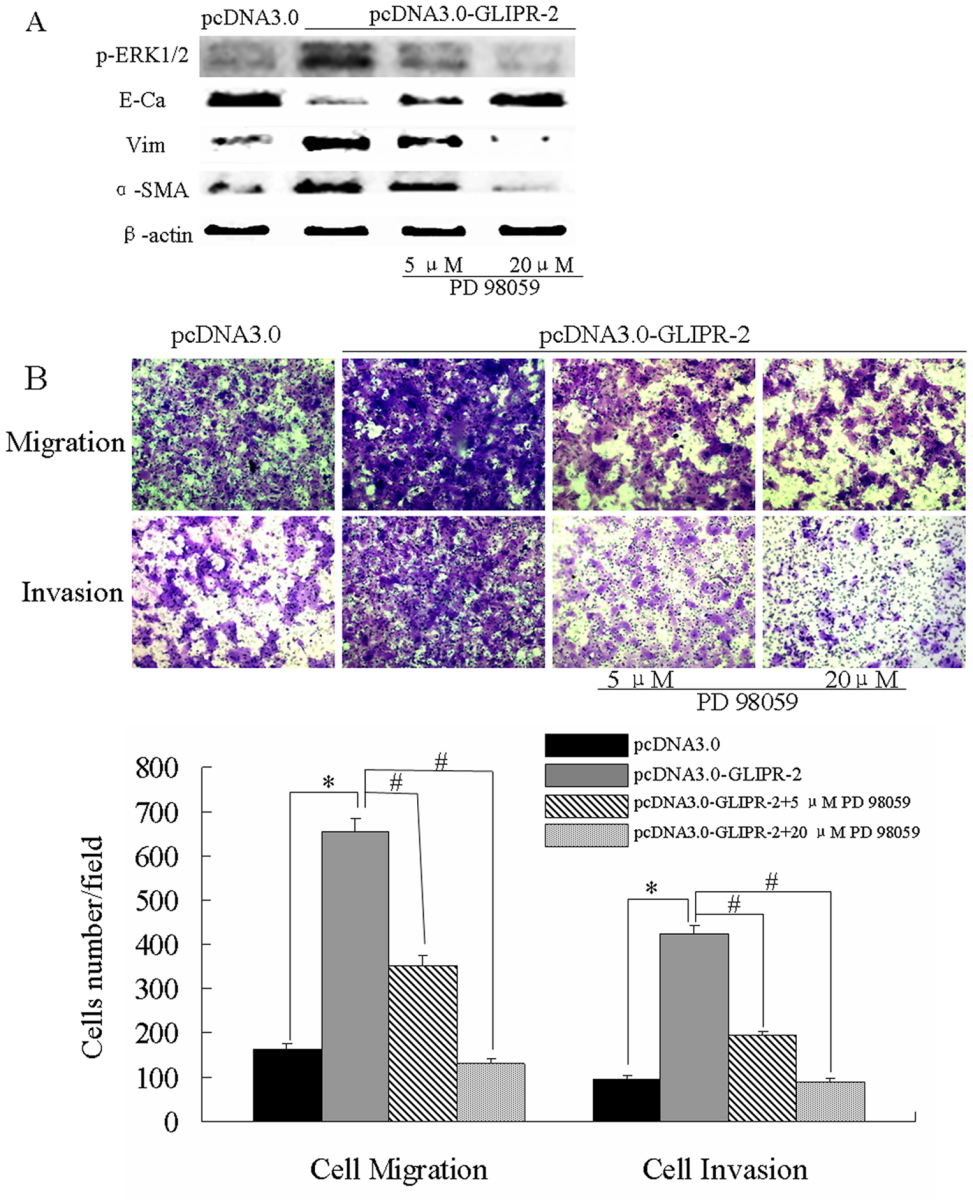
GLIPR-2 induces the EMT-like phenotype following enhanced migration and invasion of *PLC*/PRF/5 cells via ERK1/2. (A) P-ERK1/2 and EMT markers changed at equal with HepG2 data. (B) GLIPR-2 overexpession promotes cell migration and invasion via ERK1/2 activation. Data are presented as mean ± SD. *P<0.01 compared with the pcDNA3.0 group (black bar), ANOVA. ^#^ *P*<0.01 compared with the GLIPR-2 transfection group (grey bar), ANOVA.

To investigate the role of GLIPR-2 in EMT-like process induced by hypoxia, we generated GLIPR-2 overexpressed HepG2 and *PLC*/PRF/5 cells by transient transfection of the plasmid pcDNA3.0- GLIPR-2(Figure S1 A–D). We examined the expression of EMT markers and whether ERK1/2 signal pathway was activated in transfected cells. As shown in Figure 2A and Figure 3A, the E-cadherin expression level decreased after GLIPR-2 over-expression whereas vimentin and α-SMA increased. P-ERK1/2 was also upregulated in GLIPR-2 transfected groups. The ERK1/2 activation was reversed in a dose-dependent manner by an ERK inhibitor PD98059 (Figure 2 A and Figure 3A). Meanwhile, the EMT-like phenotype (5, 20 µM) was also reversed by PD98059 (Figure 2 A and Figure 3A). We further examined whether GLIPR-2 overexpression could potentiate cell migration and invasion in the two cell lines. As shown in Figure 2 B and Figure 3B, the cells with GLIPR-2 overexpression migrated faster than the control cells. These data suggested that the GLIPR-2 overexpression by recombinant plasmid promoted EMT-like process following by the migration and invasion in HepG2 and *PLC*/PRF/5 cells.

### Suppression of ERK1/2 Activation Attenuates EMT-like Process following by Migration and Invasion Induced by Hypoxia

**Figure 4:**
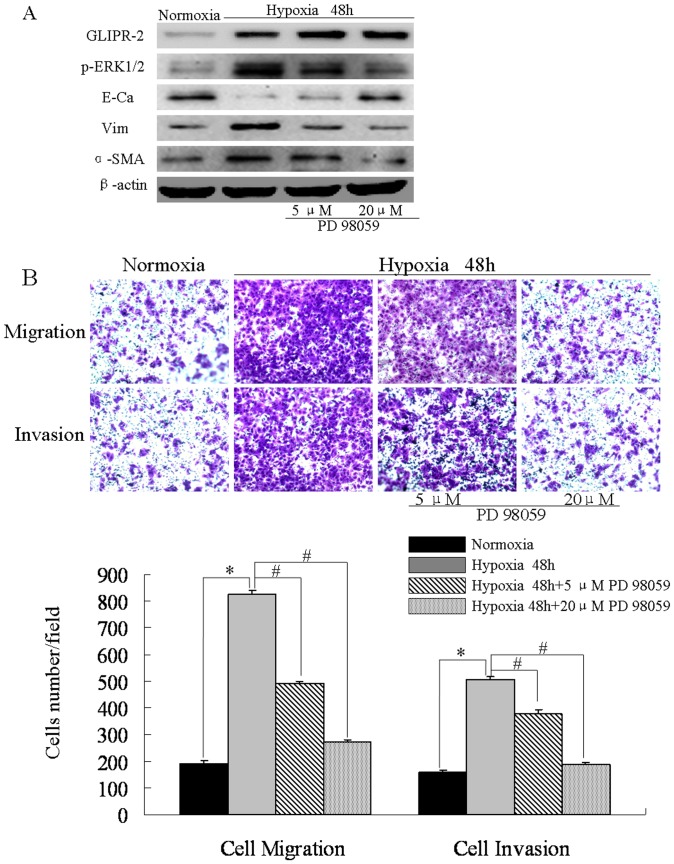
Suppression of ERK1/2 activation in human HCC cell attenuates EMT-like process following by migration and invasion induced by hypoxia. (A) GLIPR-2, p-ERK1/2 and EMT markers expression in HepG2 cells after hypoxia 48 h or treated with 5,20 µM PD 98059. GLIPR-2 expression was elevated after hypoxia 48 h. (B) Hypoxia promotes HepG2 cells migration, invasion via ERK1/2 activation. Data are presented as mean ± SD. *P<0.01 compared with the normoxia group (black bar), ANOVA. ^#^ *P*<0.01 compared with the hypoxia group (grey bar), ANOVA.

Hypoxia is known to activate MAPK/ERK1/2 pathway which induces EMT-like phenotype in human HCC cell [16], [17]. To determine whether hypoxia induce EMT in HepG2 cell line, we detected the EMT markers, E-cadherin, vimentin and α-SMA after 48 h of hypoxia. As shown in Figure 4 A, the E-cadherin protein level was significantly lower, whereas the level of GLIPR-2, vimentin and α-SMA was significantly higher in hypoxia group. Furthermore, HepG2 cells motility and invasiveness increased after hypoxia 48 h. We set out to determine whether elevated p- ERK1/2 is required for hypoxia-induced EMT.PD98059(5, 20 µM) was added into medium of HepG2 cells in hypoxic conditions (1% O_2_).As shown in Figure 4 A, p- ERK1/2 level was elevated in the HepG2 cells after 48 h hypoxic compared to normoxia, but decreased in PD98059 groups. Concurrently, E-cadherin was up-regulated whereas vimentin and α-SMA downregulated in PD98059 groups after hypoxia 48 h. PD98059 also attenuated migration and invasion induced by hypoxia in HepG2 cells (Figure 4 B). These data suggested that ERK1/2 activation was elevated in human HCC cell and suppression of ERK1/2 activation may attenuate EMT-like process following by migration and invasion induced by hypoxia.

### Suppression of GLIPR-2 Expression Attenuates ERK1/2 Activation, EMT-like Process following by Migration and Invasion in Response to Hypoxia

**Figure 5:**
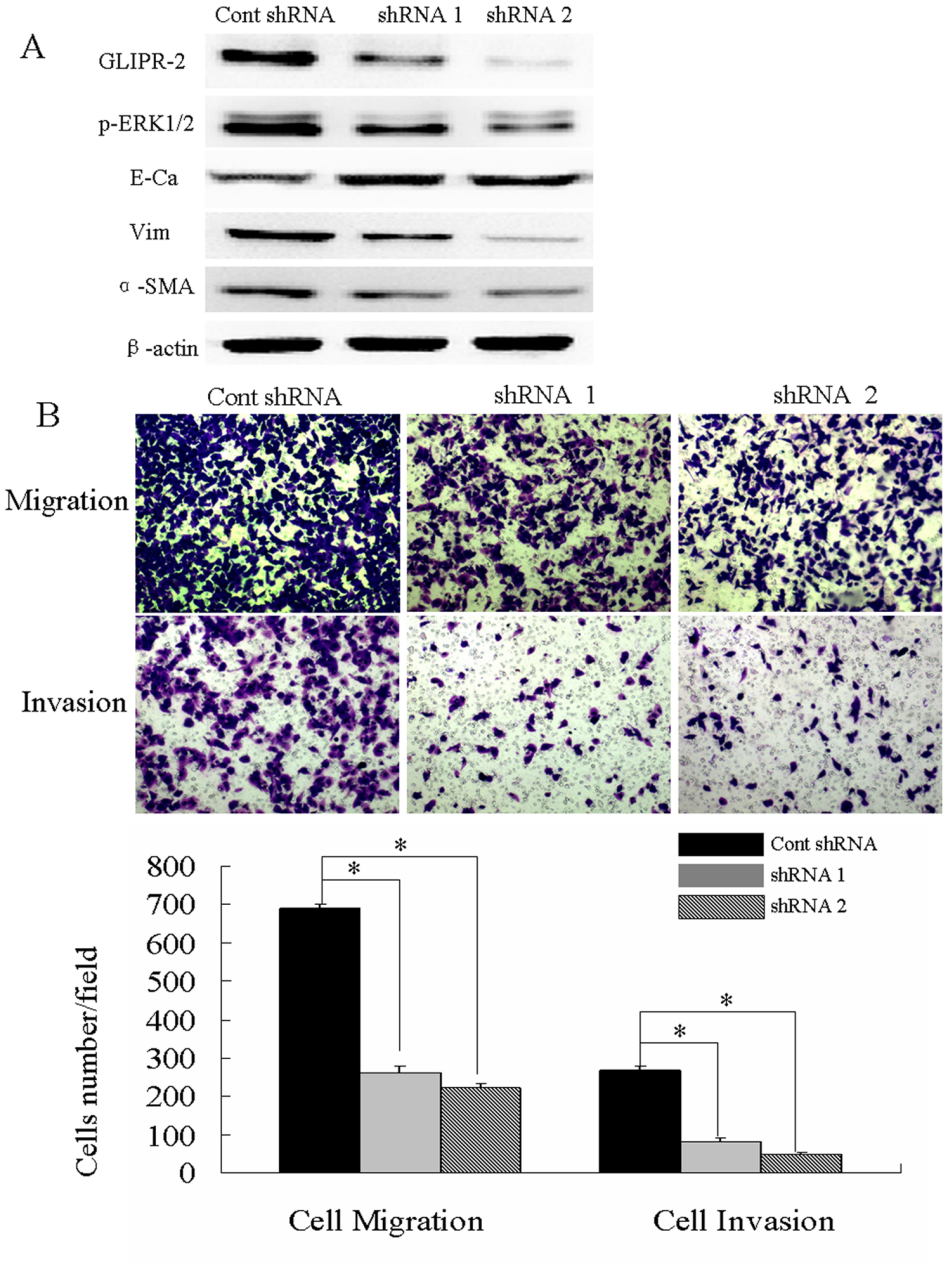
Suppression of GLIPR-2 expression attenuates ERK1/2 activation, EMT-like phenotype following by migration and invasion in response to hypoxia. (A) GLIPR-2, p-ERK1/2 and EMT markers expression in HepG2 and *PLC*/PRF/5 cells after suppression of GLIPR-2 after hypoxia 48 h. GLIPR-2 expression was efficiently suppressed by the shRNAs targeting GLIPR-2 and p-ERK1/2 decreased in HepG2 and *PLC*/PRF/5 cells in hypoxia condition. E-cadherin increased in the GLIPR-2 shRNA group but vimentin and α-smooth muscle actin decreased. (B) Suppression of GLIPR-2 expression attenuates HepG2 and *PLC*/PRF/5 cells migration and invasion. Data are presented as mean ± SD. *P<0.01 compared with the cont shRNA group (black bar), ANOVA.

To examine the role of GLIPR-2 expression in hypoxia-induced EMT and following migration and invasion, the lentiviral shRNA-GLIPR-2 were infected in HepG2 cells. After flow cytometric analysis, cells were incubated in hypoxia for 48 h (Figure S2). The knockdown efficiency has been quantified by qPCR (Figure S3). As shown in Figure 5 A, GLIPR-2 expression was suppressed efficiently in the shRNA-GLIPR-2 groups as well as p-ERK1/2. E-cadherin was upregulated whereas vimentin and α-SMA was downregulated after suppression of GLIPR-2.Cells motility and invasiveness were also decreased after suppression of GLIPR-2 in hypoxia (Figure 5 B). These results revealed that suppression of GLIPR-2 expression in human HCC cells attenuated ERK1/2 activation and EMT-like process following by migration and invasion in response to hypoxia.

## Discussion

Our studies demonstrate that GLIPR-2 is expressed in cancer tissues of hepatic carcinoma and can be induced in HCC cell lines in hypoxia condition in vitro. GLIPR-2 overexpression in HCC cells promotes migration and invasion via EMT-like changes and these changes require the activation of ERK1/2 signaling pathway.

GLIPR-2 was first found in acquisition of resistance by plants against viral infections and has been reported widely expressed in the mammalian and plant [13], [18], [19]. Although it is implicated in human brain tumor growth and kidney fibrosis, the precise biological activity remains unknown [14], [20]. Our previous studies have shown that GLIPR-2 was highly expressed in proximal renal tubular epithelial cells in diabetic nephropathy and overexpression of GLIPR-2 in HK-2 cells promotes EMT in vitro via ERK1/2 signaling pathway, which was classified as type 2 EMT involved in renal fibrosis [15]. Therefore, we hypothesized that GLIPR-2 is elevated in the EMT process in carcinogenesis (type 3 EMT) and involved in tumor invasion and metastasis.

As a micro-environmental factor known to promote tumor angiogenesis and induce EMT, hypoxia is related to treatment resistance and increased metastatic potential in solid tumors, such as hepatic carcinoma [21]. In the cancer tissues, rapid growth of cancer cells often creates insufficient supply of oxygen and results to hypoxic microenvironment. In this study, we found that GLIPR-2 expression increased in HCC cells partially in the liver cancer paraffin-embedded tissue sections. Similar to this result, we found increased GLIPR-2 expression in HCC cell lines under hypoxia conditions in vitro. Due to the complexity of carcinogenesis, other factors may also upregulate GLIPR-2 expression such as cytokines, which need further investigation. To investigate the function of GLIPR-2, we detected the motility and invasiveness of GLIPR-2 overexpression HCC cells. Our data suggested that overexpression of GLIPR-2 in HCC cells promoted migration and invasion. These data supports our previous hypothesis and may hint the relationship between GLIPR-2 expression and EMT.

EMT has been thought to promote both metastatic progression of cancer and acquisition of stem-cell characteristics in carcinogenesis, leading to poorer patient survival [3], [22], [23]. Various growth factors including transforming growth factor-β1, hepatocyte growth factor, and platelet-derived growth factor could induce EMT in vitro, however, the mechanisms by which EMT generate remain to be elucidated. Interestingly, the data showed that GLIPR-2 overexpression induces an EMT-like cell phenotype change in HCC cells. We for the first time report expression and function of GLIPR-2 in HCC cell lines. Previous studies have shown that Ras/ERK-MAPK signaling has been shown to participate in EMT [11]. Hypoxia could increase p-ERK1/2 level and activation of ERK-1/2 in tumor cells has been shown to be associated with enhanced migration and invasion [24], [25], [26]. In this study, we found that hypoxia promoted phosphorylation of ERK1/2 and blockade of the activation with PD98059 reversed EMT-like process and migration and invasion, suggesting that ERK1/2 pathways contributed to EMT. In our previous studies, GLIPR-2 has shown the association with EGFR -mediated signaling. Similarly, we also found that GLIPR-2 upregulated p-ERK1/2 levels and contributed to EMT. Moreover PD98059 inhibit the migration and invasion and EMT-like changes enhanced by GLIPR-2 expression in HCC cells. To further define the mechanism of the increased migration and invasion by GLIPR-2, we detect whether inhibition GLIPR-2 could attenuate ERK1/2 activation, EMT-like process following by migration and invasion in response to hypoxia in human HCC cells. Similarly, we found that blockade GLIPR-2 expression in hypoxia decreased p-ERK1/2 levels and EMT-like process following by migration and invasion. Taken together, the data suggest that GLIPR-2 expression promotes migration and invasion of HCC cells via EMT through ERK1/2 activation.

**Figure 6:**
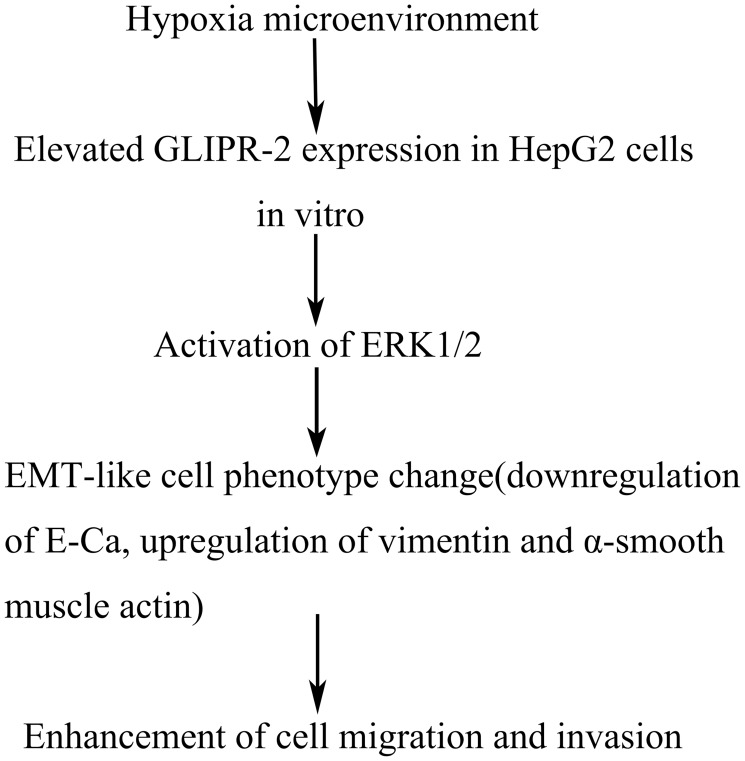
Schematic representation of GLIPR-2 induced by hypoxia promoting cell migration and invasion via EMT through ERK1/2 activation in HCC cells in vitro.

In conclusion, we demonstrate that GLIPR-2 expressed in HCC tissues and could be induced by hypoxia in HCC cell lines in vitro. In addition, GLIPR-2 overexpression in HepG2 cells promotes EMT-like process following by enhanced migration and invasion and this process need activation of ERK1/2. In contrast, suppression of GLIPR-2 expression could attenuate EMT-like process following by migration and invasion via ERK1/2 activity in response to hypoxia (Figure 6). Our results suggest that GLIPR-2 could be used as a potential therapeutic target of tumors with hyperactive ERK1/2 signaling pathway. However, further work is needed to determine the extrapolation of in vitro results to an in vivo situation.

## Supporting Information

Figure S1 **GLIPR-2 expression in pcDNA3.0- GLIPR-2-transfected HepG2 and** ***PLC*** **/PRF/5 cells.** (A, C) GLIPR-2 mRNA expression in pcDNA3.0 (mock vector) and pcDNA3.0-GLIPR-2-transfected HepG2 and *PLC*/PRF/5 cells. (B, D) GLIPR-2 protein (18 kDa) expression in pcDNA3.0 (mock vector) and pcDNA3.0-GLIPR-2-transfected HepG2 and *PLC*/PRF/5 cells.
(TIF)

Figure S2 (A) Fluorescent detection of control shRNA and GLIPR-2 shRNAs in HepG2 cells 48 hours post-transfection, demonstrating high transfection efficiency. (B) Flow cytometry analysis of transfented HepG2 cells showed the EGFP-positive population was selected.
(TIF)

Figure S3 **QRT-PCR data of knockdown efficiency in HepG2 cells on GLIPR-2.** GLIPR-2 expression decreased in shRNA1 and shRNA2 groups. Data are presented as mean ± SD. *P<0.01 compared with the cont shRNA group, ANOVA.
(TIF)
