# Supplementary figures and images for: Hypoxia Promotes Epithelial - Mesenchymal Transition of Hepatocellular Carcinoma Cells via Inducing GLIPR-2 Expression
Source: PLoS One. 2013 Oct 29;8(10):e77497. doi: 10.1371/journal.pone.0077497 (PMC3812270; doi:10.1371/journal.pone.0077497)

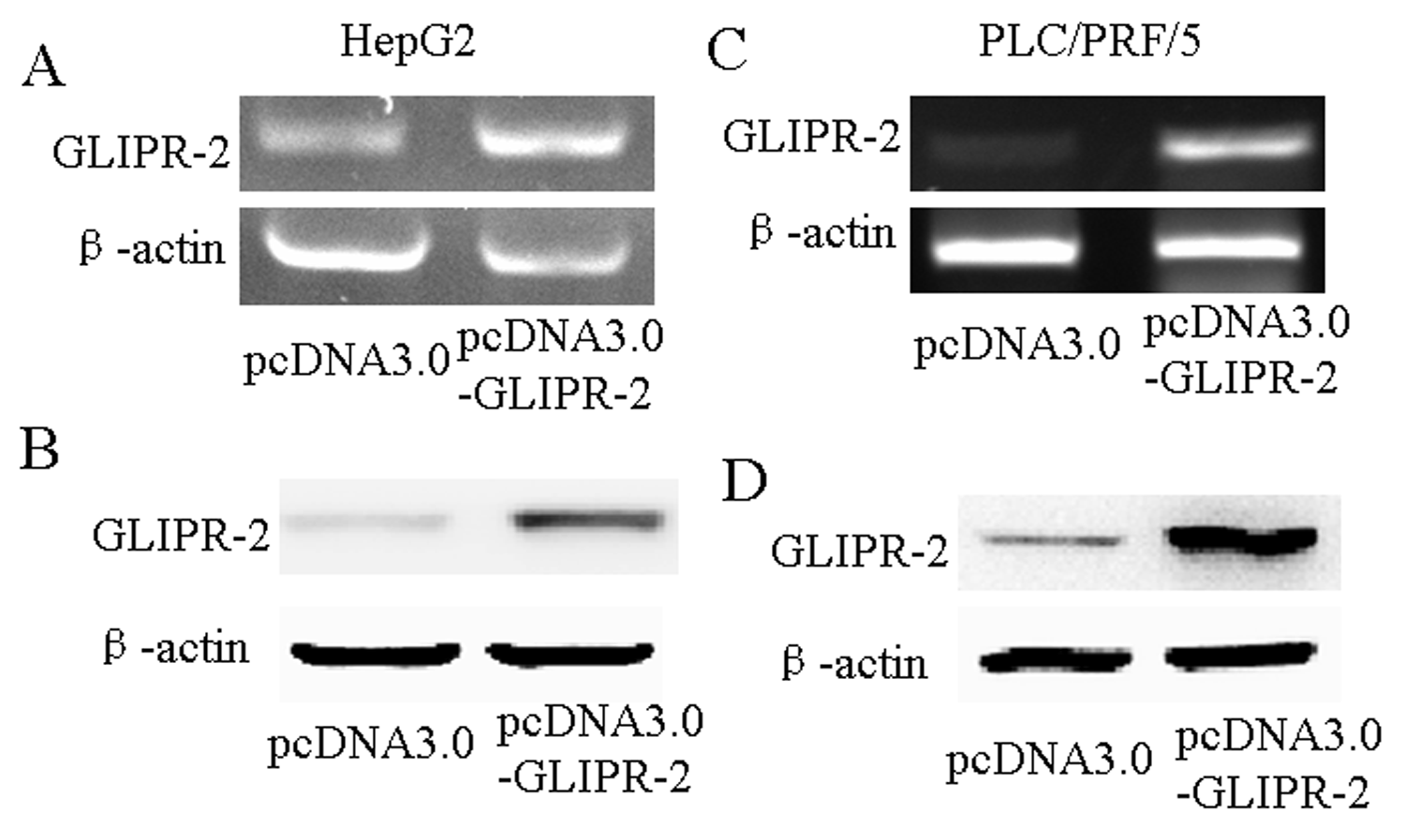

Supplement: Figure S1 — GLIPR-2 expression in pcDNA3.0- GLIPR-2-transfected HepG2 and PLC /PRF/5 cells. (A, C) GLIPR-2 mRNA expression in pcDNA3.0 (mock vector) and pcDNA3.0-GLIPR-2-transfected HepG2 and PLC/PRF/5 cells. (B, D) GLIPR-2 protein (18 kDa) expression in pcDNA3.0 (mock vector) and pcDNA3.0-GLIPR-2-transfected HepG2 and PLC/PRF/5 cells. (TIF) [file pone.0077497.s001.tif]

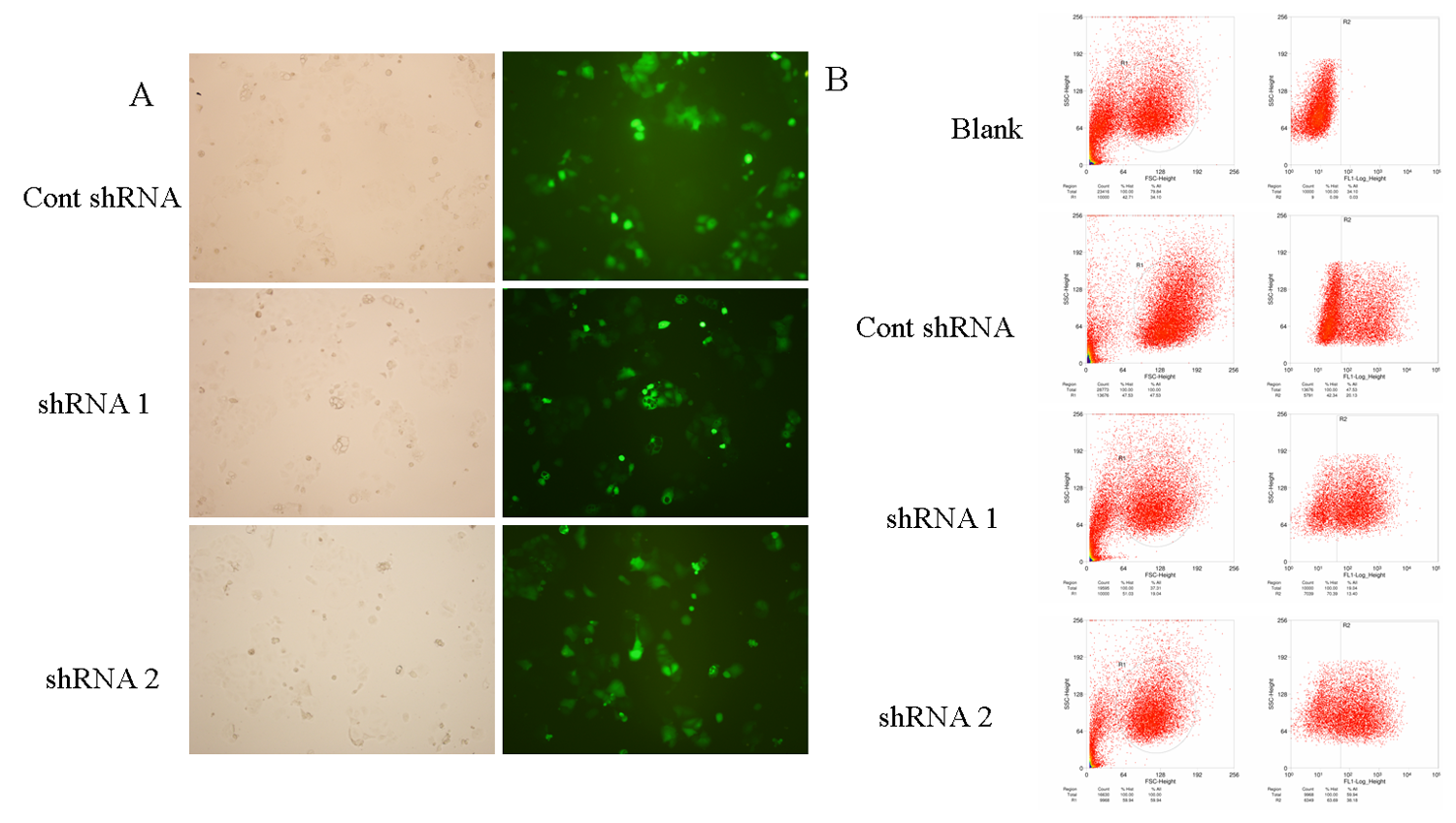

Supplement: Figure S2 — (A) Fluorescent detection of control shRNA and GLIPR-2 shRNAs in HepG2 cells 48 hours post-transfection, demonstrating high transfection efficiency. (B) Flow cytometry analysis of transfented HepG2 cells showed the EGFP-positive population was selected. (TIF) [file pone.0077497.s002.tif]

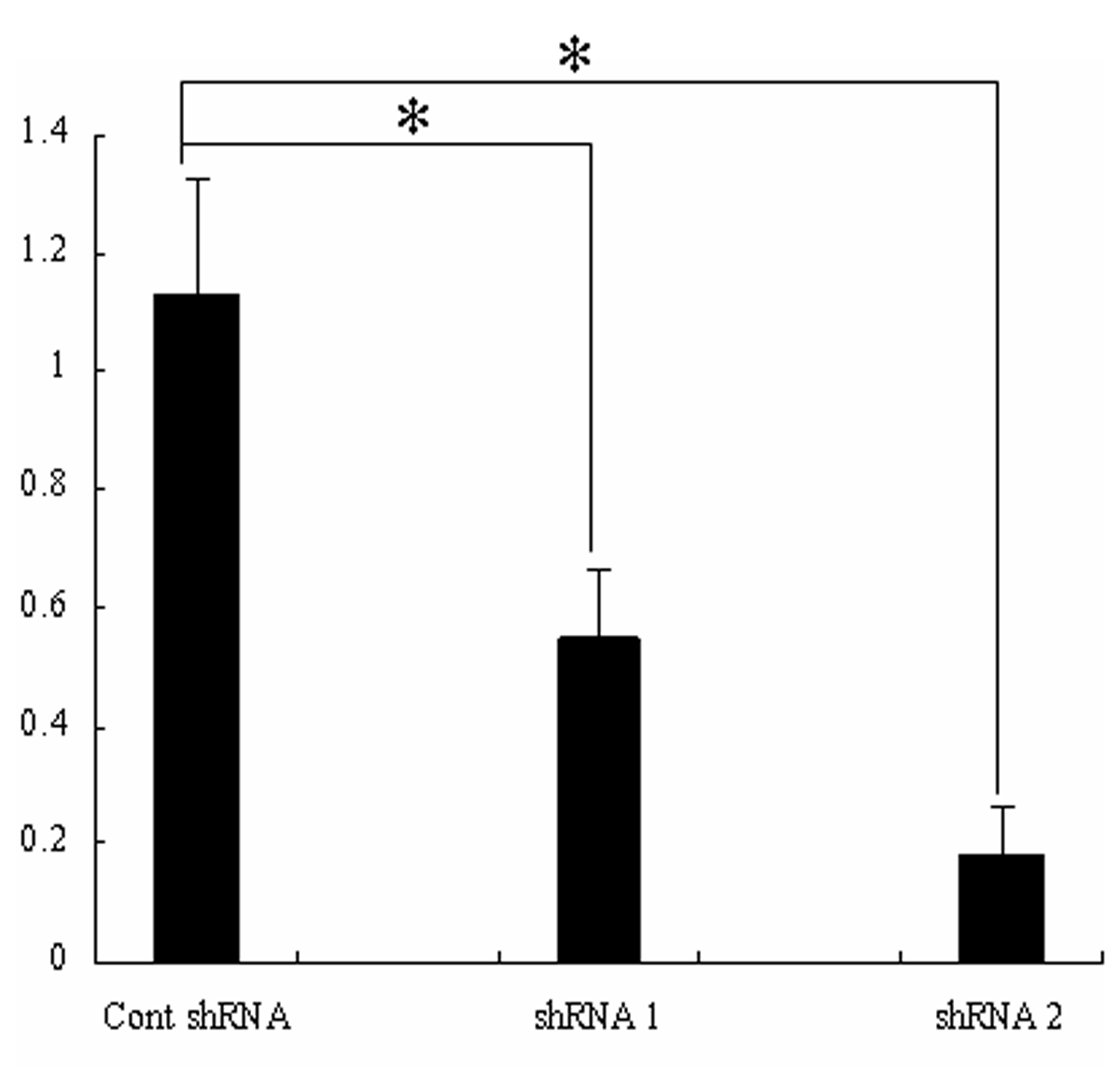

Supplement: Figure S3 — QRT-PCR data of knockdown efficiency in HepG2 cells on GLIPR-2. GLIPR-2 expression decreased in shRNA1 and shRNA2 groups. Data are presented as mean ± SD. *P<0.01 compared with the cont shRNA group, ANOVA. (TIF) [file pone.0077497.s003.tif]
